# Overdose Toxicological Effect of Methanol Extract of Popular Edible *Colocasia esculenta* Linn. Flowers: Biochemical, Hematological, and Behavioral Study on Swiss Albino Mice

**DOI:** 10.1002/fsn3.70674

**Published:** 2025-07-23

**Authors:** Mahathir Mohammad, Md. Tanvir Chowdhury, Nazmul Hasan Eshaque, Md. Jahirul Islam Mamun, Sayed Al Hossain Rabbi, Md. Amzad Hasan, Miton Chowdhury, Md. Al‐mamun, Md. Jabir Rashid, S. M. Moazzem Hossen

**Affiliations:** ^1^ Department of Chemistry Chittagong University of Engineering & Technology Chittagong Bangladesh; ^2^ Department of Pharmacy, Faculty of Biological Sciences University of Chittagong Chittagong Bangladesh; ^3^ Department of Chemistry Government City College Chittagong Bangladesh; ^4^ Department of Pharmacy University of Science and Technology Chittagong Chittagong Bangladesh; ^5^ Department of Pharmacy University of Asia Pacific Dhaka Bangladesh

**Keywords:** antidepressant, anxiolytic, *Colocasia esculenta* Linn., hematology, hepatoprotective, sedative

## Abstract

*Colocasia esculenta* Linn., an annual herbaceous plant from the Araceae family, has long been utilized in traditional medicine throughout tropical and subtropical regions for the treatment of diverse health conditions. This research explored the impact of the methanolic extract derived from *C. esculenta* flowers (CEF‐ME) on mice, examining various physiological, biochemical, hematological, and behavioral responses. Behavioral assessments for anxiolytic activity included the Elevated Plus Maze, Hole‐Board Test, and Light–Dark Box Test. Sedative properties were evaluated using the Open‐Field Test and Hole‐Cross Test, while potential antidepressant effects were analyzed via the Tail‐Suspension Test and Forced‐Swimming Test. The administration of CEF‐ME led to observable changes in organ‐to‐body weight ratios, most notably a significant increase in liver size, which may indicate an upregulation of metabolic or detoxification functions. Biochemical tests revealed a hepatoprotective trend, characterized by decreased levels of ALT, ALP, bilirubin, cholesterol, and triglycerides. Yet, elevations in AST and creatinine at higher doses suggested potential hepatic and renal strain. Hematological profiles showed enhanced immune activity and red blood cell production, though the drop in platelet counts raised concern for thrombocytopenia. Behavioral evaluations revealed the extract's anxiolytic, sedative, and antidepressant properties, with effects comparable to diazepam and fluoxetine. The therapeutic potential of CEF‐ME was evident; however, its administration at high doses triggered toxicity concerns, particularly impacting the hepatic and renal systems. These observations highlight a pressing need for further studies to investigate its prolonged safety, pharmacological mechanisms, and optimal therapeutic range.

## Graphical abstract

*Colocasia esculenta* Linn., an annual herbaceous plant from the Araceae family, has long been utilized in traditional medicine throughout tropical and subtropical regions for the treatment of diverse health conditions. This study investigated the effects of the methanolic extract of *C. esculenta* flowers (CEF‐ME) on mice, focusing on physiological, biochemical, hematological, and behavioral parameters.

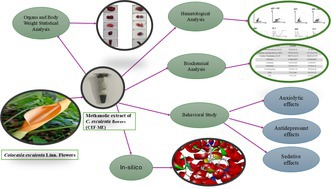

## 1 Introduction

Neurological illnesses are presently a major contributor to morbidity and disability worldwide (Chin and Vora 2014). In 2021, neurological illnesses impacted over 3 billion individuals, or around 43% of the worldwide population, underscoring the urgent necessity for improved treatment alternatives and more access to information (Harris 2024). The locomotor mechanism, commonly referred to as the musculoskeletal system, is a complex organ system that enables movement in humans and animals. A notable correlation occurs between mental health issues, particularly anxiety and depression, and musculoskeletal diseases, with affected persons frequently encountering exacerbated symptoms and prolonged recovery periods (Heikkinen et al. 2019). The financial impact of neurological disorders is substantial, surpassing the costs associated with other significant diseases such as cancer and cardiovascular conditions. In Europe, the total expense of neurological disorders was estimated at $1.06 trillion in 2020 (Tripathi et al. 2024). This financial burden affects individuals, families, communities, and entire economies.

Toxicology refers to the detrimental effects resulting from the interaction of toxicants with cells. It is a synonym for toxicology. The position of this contact may fluctuate based on the characteristics of the cell membrane and the toxicants, as well as the extracellular matrix, cell surface, cell body, or underlying tissues. Adverse effects may already manifest before toxicants reach vital organs such as the liver or kidneys. It is essential to evaluate a substance's hazardous characteristics when addressing public health protection, as chemical exposure can be detrimental and adversely affect people. Evaluations frequently encompass carcinogenic, reproductive, subchronic, chronic, and acute impacts (Jothy et al. 2011). Various Categories of Hazards Acute toxicity refers to the adverse effects resulting from a singular encounter with a poisonous agent. Metrics such as LD_50_ (lethal dose for 50% of a test population) and LC_50_ (lethal concentration for 50% of a test population) are commonly employed to assess acute toxicity by SAR and QSAR modeling of an extensive dataset of LD_50_ rat acute oral toxicity information. The intensity and rapidity of the symptoms may fluctuate based on the substance and dosage. Conversely, Long‐Term Toxicology indicates that frequent or prolonged exposure to a hazardous substance may result in chronic toxicity, leading to enduring health consequences (Spielmann et al. 1999). These adverse effects may manifest progressively and could encompass severe conditions such as cancer, organ damage, or reproductive issues. The gradual onset of chronic poisoning and its mild symptoms complicate diagnosis.

*Colocasia esculenta* , often known as taro, belongs to the Arum family (Araceae). The primary cultivation of this tropical tuber crop is for its underground tubers, which are predominantly consumed in tropical locations globally (Kaushal et al. 2015). In Bangladesh, “Kachu” is the predominant designation for Taro, which is also referred to by several local names in different places. The plant is also known by various names, including cocoyam, eddoe, dasheen, tannia, keladi, and talo futuna, among other (Onwueme 1999). Ayurvedic medicine employs *C. esculenta* to address vata‐pitta imbalances, constipation, alopecia, stomatitis, hemorrhoids, and weakness (Pawar et al. 2018). Leaf juice is administered topically for scorpion stings, snake bites, and plant‐based food poisoning (Patil and Ageely 2011). Numerous investigations have shown substantial pharmacological activity in various components of *C. esculenta* . The leaves and corms of *C. esculenta* demonstrate hepatoprotective properties against liver damage (Jain et al. 2023). The root extract of *C. esculenta* demonstrates considerable neuroprotective benefits and anti‐inflammatory properties in both in vitro and in vivo tests (Khazaal et al. 2024). The leaves of *C. esculenta* have antibacterial properties against *Staphylococcus aureus* , *Pseudomonas aeruginosa* , and other pathogens, which are ascribed to the existence of advantageous secondary metabolites in the leaves (Shelke et al. 2024). The leaves of *C. esculenta* were reported to have notable antifungal properties (Patil and Ageely 2011). Hydroalcoholic extracts of *C. esculenta* leaves demonstrate anxiolytic, depressive, and sedative properties, although they may compromise motor coordination at specific dosages, necessitating careful evaluation for therapeutic application (Kalariya et al. 2010).

This study aimed to evaluate the toxicological and neuropharmacological characteristics of methanolic extracts from *C. esculenta* flowers, considering both conventional applications and existing data from several experimental models.

## 2 Materials and Methods

### 2.1 Chemicals

Analytical‐grade reagents and chemicals were utilized during the experiments.

### 2.2 Plant Collection, Identification, and Extraction

*Colocasia esculenta* flowers were collected from Fulgazi, Feni, Chattogram, Bangladesh, in August 2022. The species was identified by Mr. Md. Owahidul Alom, Department of Botany, University of Chittagong. A voucher specimen (no. DP/CU/2023/011) is preserved for future reference.

### 2.3 Experimental Animal and Experimental Design

In this investigation, healthy male Swiss albino mice (25–35 g, 4–5 weeks old) from BCSIR Chittagong were housed at USTC Pharmacy under controlled conditions, given water and a standard diet, acclimated for a week, fasted for 12 h before the trial, and studied in a soundproof environment with USTC ethics approval (USTMEBBC/23/09/04).

#### 2.3.1 Animal Distribution

For pharmacological evaluation, the mice were randomly allocated into four groups.

- *Group I* (Negative Control): Received only the vehicle to establish a baseline for comparison.
- *Group II* (Positive Control): Treated with a standard drug to serve as a reference.
- *Group III* (low‐dose extract): Received CEF‐ME at a dose of 200 mg/kg body weight.
- *Group IV* (high‐dose extract): Received CEF‐ME at a dose of 400 mg/kg body weight.

The therapeutic dose was calculated as one‐tenth of the median lethal dose (LD_50_ > 2.0 g/kg) using Karber's arithmetic method along with the Hodge and Sterner scale. The chosen dose for this study is based on the results of the acute toxicity study. Since one‐tenth of 2000 mg/kg is 200 mg/kg, this dose was applied in the experiment. Furthermore, a higher dose of 400 mg/kg, which is double the initial dose, was included to assess the extract's dose‐dependent effects.

### 2.4 Acute Toxicity Assay

Mice were housed under controlled conditions (25°C, 12‐h light/dark cycle) with tail markings for identification and a 1‐week acclimation period. Following OECD guidelines, they received either methanolic *C. esculenta* extract (2000 mg/kg) or a saline control, with daily monitoring for 14 days to assess toxicity, behavior, and physical changes. The therapeutic dose was calculated as one‐tenth of the median lethal dose (LD_50_), following Karber's arithmetic method (Hackam and Anand 2003), and aligned with the Hodge and Sterner toxicity classification (LD_50_ > 2.0 g/kg) (Zaoui et al. 2002). The LD_50_ value was obtained using the following formula:

$$LD_{50}=LD_{100}-\sum \left(a\times b\right)/n$$

Here, *n* indicates the total number of animals in each group, *a* is the difference in dose between two consecutive treatments with the extract or substance, b shows the average number of deaths between two consecutive doses, and LD_100_ represents the dose that causes 100% mortality in the test animals.

### 2.5 Organs and Body Weight Statistical Analysis

Fourteen days later, every mouse was sacrificed. The main organs, including the kidneys, liver, lungs, spleen, and heart, were removed and examined for anomalies. Every organ was weighed, and the attributes of the treatment and control groups were compared.

$$\text{Organ body index}=\left(\text{Organ Weight}/\text{Total Body Weight}\right)\times 100$$

### 2.6 Hematological Analysis

#### 2.6.1 Blood Biomarker Assay

After 2000 mg/kg CEF‐ME extract was administered to the mice for 14 days, the mice were sacrificed, and their blood was collected in a vacutainer for biochemical analysis. Analyze the serum for lipid profile (CHO, TG, HDL, and LDL), renal function (creatinine), and liver function tests (ALT, AST, ALP, and bilirubin).

### 2.7 Anxiolytic Activity

#### 2.7.1 Elevated Plus‐Maze Test

This validated study assessed the anxiolytic effects in mice using a raised plus‐maze, where three groups (test, positive control, negative control) received test extracts (200 and 400 mg/kg), diazepam (1 mg/kg), or vehicle. Their arm entries were recorded over a 120‐min period in a sound‐insulated environment (Islam et al. 2025).

#### 2.7.2 Hole‐Board Test

The hole‐board apparatus, used to assess neophilia and anxiety in mice via head‐dipping behavior, was employed by placing mice individually for 30 min before administering control, standard, and test samples, then recording head‐dip frequency over 5 min using a tally counter (Islam et al. 2025).

#### 2.7.3 Light–Dark Box Test

The light–dark box test, adapted from a previous method, assessed the anxiolytic efficacy of CEF‐ME using a 18 × 18 × 18 cm^3^ apparatus with a 400 lx light source, where randomly assigned mice were treated, placed in the dark chamber after 30 min, and observed for transitions and time spent in light over 5 min. (Ali et al. 2025)

### 2.8 Antidepressant Activity

#### 2.8.1 Forced‐Swimming Test

This study evaluated the antidepressant effect of the experimental drug using a 25 cm × 15 cm × 25 cm water‐filled container (25°C ± 1°C, 19 cm depth), where each mouse was placed alone for 5 min, and immobility duration, defined as the time spent without struggling while keeping its head above water, was recorded (Ali et al. 2025).

#### 2.8.2 Tail‐Suspension Test

With minor modifications, this test involved six groups of five mice each, where Groups III–VI received *C. esculenta* extracts (200 and 400 mg/kg, p.o.), a negative control (1% Tween‐80 in saline, 10 mL/kg), or a positive control (Fluoxetine, 25 mg/kg). Thirty minutes post‐administration, mice were suspended 50 cm above the floor using adhesive tape, and immobility, defined as hanging silently or staying still, was recorded for 6 min (Ali et al. 2025).

### 2.9 Sedative Activity

#### 2.9.1 Open‐Field Test

The 0.5 m^2^ apparatus, enclosed by 50 cm walls with a checkered floor, was used to track mice movement post‐oral administration at 0, 30, 60, 90, and 120 min for control (saline), standard (diazepam), and test groups (200 and 400 mg/kg), recording squares crossed over 3 min using a tally counter (Ali et al. 2025).

#### 2.9.2 Hole‐Cross Test

The study took place in a 30 × 20 × 14 cm wooden room, divided by a frame with a 3.5 cm diameter opening. The frequency of mice passing through the opening was recorded at 0, 30, 90, and 120 min over a 3‐min period (Nath et al. 2025).

### 2.10 In Silico Computer‐Aided Virtual Screening

#### 2.10.1 Software Tools

The study utilized UCSF Chimera, AutodockVina, Discovery Studio Visualizer 2020 (BIOVIA), DrugBank, MGL Instruments, Protein Data Bank (PDB), and PubChem.

#### 2.10.2 Selection of the Target Proteins

A comprehensive review of current research compiled potential therapeutic targets for anxiety, depression, and sedation, which were then revalidated using the PDB of large biological molecules (Goodsell et al. 2019).

#### 2.10.3 Preparation of Target Proteins

Human GABA_A_ receptor alpha1‐beta2‐gamma2 (PDB: 6X3T), Human metabotropic GABA(B) receptor (PDB: 6UO8), Gamma‐aminobutyric acid receptor (PDB ID: 4COF), Human monoamine oxidase A (PDB ID: 2Z5X), 5‐HT1B‐BRIL receptor (PDB: 4IAQ), and Paroxetine‐human serotonin transporter complex (PDB: 5I6X) were retrieved from the RCSB PDB and used in the study, ensuring reproducibility of findings, with protein ligands removed and proteins optimized for docking using Discovery Studio Visualizer (BIOVIA) and UCSF Chimera. 6X3T, 6UO8, 2Z5X, 4COF, 5I6X, and 4IAQ are validated receptors for activated sedation, anxiety, and depression.

#### 2.10.4 Virtual Filtering

The virtual screening was conducted using PyRx‐AutodockVina software (Masters et al. 2020).

### 2.11 Statistical Analysis

The results were expressed as mean ± SEM, with statistical analysis performed using SPSS (version 25) for ANOVA, followed by a post hoc Dunnett test, with significance set at *p < 0.05, **p < 0.01, and ***p < 0.001.

## 3 Results

### 3.1 Toxicity Profile

#### 3.1.1 Acute Toxicity Study

Tables 1 and 2, respectively, illustrate the effects of *C. esculenta* flower extract in methanol on the general behavior patterns and appearance of mice.

**TABLE 1:** Acute toxic effects of the crude extract of *Colocasia esculenta* flowers in mice.

Observation	Control (treatment without crude extract)	Test group A (treatment with 2000 mg/kg)
Number of dead mice/number of mice used	0/5	0/5

**TABLE 2:** General appearance and behavioral observations for control and treated groups during the acute toxicity study.

Observation	Control group		2000 mg/kg		4000 mg/kg	
	6 h	14 h	6 h	14 h	6 h	14 h
Skin and fur	Normal	Normal	Normal	Normal	Normal	Normal
Eyes	Normal	Normal	Normal	Normal	Normal	Normal
Mucous membrane	Normal	Normal	Normal	Normal	Normal	Normal
Behavioral patterns	Normal	Normal	Rapid heartbeat	Normal	Rapid heartbeat	Normal
Salivation	Normal	Normal	N.O	N.O	N.O	N.O
Lethargy	Normal	Normal	N.O	N.O	Observed	N.O
Sleep	Normal	Normal	N.O	N.O	N.O	Observed
Diarrheal	Normal	Normal	N.O	N.O	N.O	N.O
Coma	N.O	N.O	N.O	N.O	N.O	N.O
Tremors	N.O	N.O	N.O	N.O	N.O	N.O

#### 3.1.2 Organ and Body Weight Statistical Analysis

**FIGURE 1:**
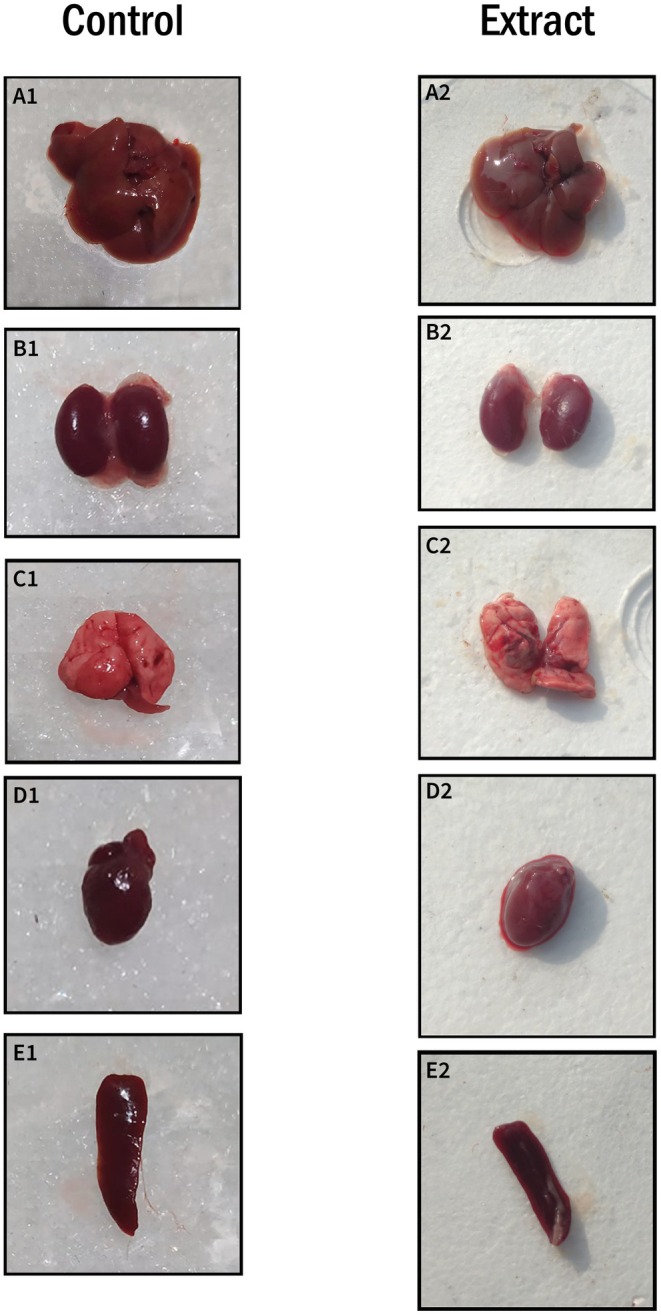
Gross observation of systemic organs: Liver (A1 and A2), kidney (B1 and B2), lung (C1 and C2), heart (D1 and D2), and spleen (E1 and E2) from control and extract‐treated mice.

Crude methanolic extract of *C. esculenta* flowers was administered to mice at a dose of 2000 mg/kg; values are mean ± SD (*n* = 3) at the 5% level of significance (*p < 0.05). The gross observations of systemic organs from control and extract‐treated mice are shown in Table 3 and Figure 1.

**TABLE 3:** Effect of CEF‐ME on organ‐to‐body weight index (%) in mice. CEF‐ME = *Colocasia esculenta* flowers methanol extract.

Organ	Body and organ weight index of mice before and after treatment of 14 days	
	Control	Treatment
Liver	5.44 ± 0.35	8.81 ± 0.49*
Lung	0.91 ± 0.09	1.31 ± 0.059*
Kidney	1.47 ± 0.1	2.58 ± 0.41***
Heart	0.67 ± 0.07	1.11 ± 0.11*
Spleen	0.47 ± 0.04	0.80 ± 0.15**

*Note:* Statistical significance levels were denoted as follows: *p < 0.05, **p < 0.01, and ***p < 0.001, in comparison to the control group.

#### 3.1.3 Biochemical Analysis

Table 4 presents the biochemical evaluation of the extract CEF‐ME and its effects on various organs. The table provides detailed insights into the biochemical parameters assessed, which include markers of liver function (e.g., ALT, AST, ALP), kidney function (e.g., creatinine, urea), lipid profiles (e.g., cholesterol, triglycerides), and other relevant indicators depending on this study design.

**TABLE 4:** Biochemical report of liver function tests, renal function tests, and lipid profile tests.

Biochemical parameters	Control	Treatment
Alanine aminotransferase (ALT)	54.43 ± 6.54	39.46 ± 3.1
Aspartate aminotransferase (AST)	370.83 ± 10.20	421.73 ± 17.87
Alkaline Phosphatase (ALP)	490.73 ± 18.30	384.23 ± 19.70
Bilirubin	0.066 ± 0.003	0.046 ± 0.003
Creatinine	0.42 ± 0.026	0.69 ± 0.07
Cholesterol	139.63 ± 5.13	67.26 ± 6.41
Triglycerides	175.9 ± 9.18	79.43 ± 9.60
HDL	77.5 ± 4.86	44.13 ± 4.25
LDL	17.2 ± 1.81	11.63 ± 1.69

#### 3.1.4 Hematological Study

**FIGURE 2:**
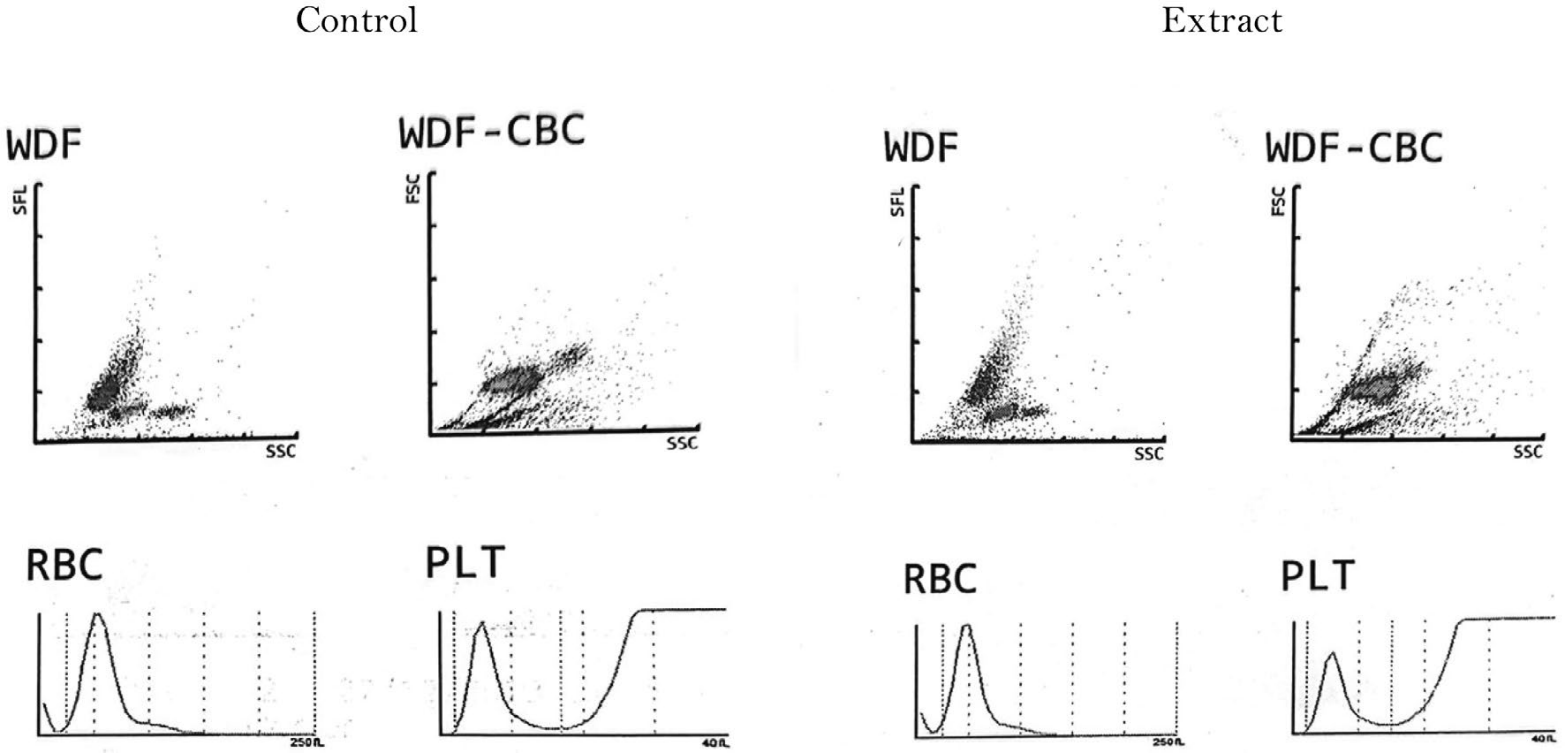
Effect of extract CEF‐ME on multiple hematological parameters. CEF‐ME = *Colocasia esculenta* flowers methanol extract.

Table 5 and Figure 2 present the hematological evaluation of the extract CEF‐ME and its effects on various blood parameters.

**TABLE 5:** Effect of single oral administration of the plant extract (2000 mg/kg) on hematological parameters.

Hematological parameter	Control	Treatment
White blood cell (10^3^/μL)	7.92 ± 0.53	8.72 ± 0.51
Red blood cell (10^6^/μL)	8.1 ± 0.58	9.14 ± 0.03*
Hemoglobin (g/dL)	12.06 ± 0.35	13.06 ± 0.31
Hematocrit blood test (%)	45.6 ± 3.60	43.93 ± 1.16
Mean cell hemoglobin (pg)	15.06 ± 0.72	14.4 ± 0.26
Mean corpuscular hemoglobin concentration (g/dL)	26.86 ± 2.44	29.7 ± 1.57*
Platelet count test (10^3^/μL)	1020 ± 104.84	751 ± 11.15*
Red cell distribution width standard deviation (fL)	35.53 ± 2.69	33.46 ± 1.86
Red cell distribution width coefficient of variation (%)	21.6 ± 1.17	21.83 ± 1.15
Platelet distribution width (fL)	12.56 ± 3.10	16.36 ± 5.85
Mean platelet volume (fL)	6.9 ± 0.05	6.9 ± 0.05
Platelet large cell ratio (%)	5.93 ± 0.86	6.7 ± 0.20
Platelet care technician (%)	0.70 ± 0.07	0.521 ± 0.005
Neutrophil (%)	18.66 ± 2.01	24.56 ± 7.69*
Lymphocyte (%)	74.53 ± 3.77	53.8 ± 13.20*
Monocyte (%)	1.2 ± 0.32	4.66 ± 1.20**
Eosinophil (%)	0.53 ± 0.27	0.83 ± 0.46
Basophil (%)	4.06 ± 2.33	7.33 ± 4.53
Immunoglobulin (%)	0.066 ± 0.033	0.16 ± 0.06*

*Note:* Statistical significance levels were denoted as follows: *p < 0.05, **p < 0.01, and ***p < 0.001, in comparison to the control group.

### 3.2 Anxiolytic Activity

#### 3.2.1 Elevated Plus‐Maze Test

**FIGURE 3:**
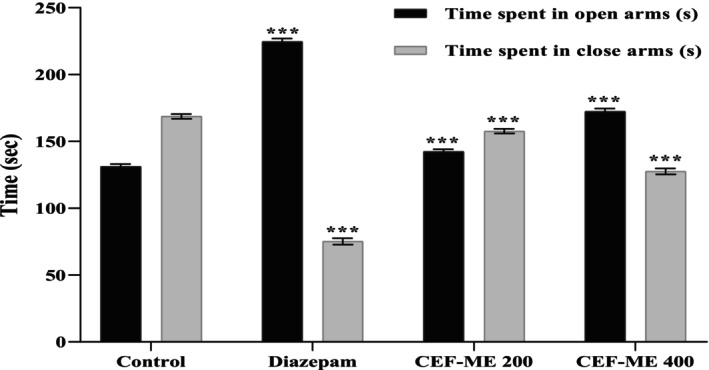
Evaluation of anxiolytic activity of CEF‐ME through the use of EPM. CEF‐ME = *Colocasia esculenta* flowers methanol extract.

CEF‐ME showed dose‐dependent anxiolytic properties in this test. When compared to the control, CEF‐ME at both 200 and 400 mg/kg spent a greater amount of time in the open arms, as shown in Table 6 and Figure 3.

**TABLE 6:** Effects of CEF‐ME on mice in the elevated plus maze apparatus. CEF‐ME = *Colocasia esculenta* flowers methanol extract.

Test samples	Dose (mg/kg)	Time spent in open arms (s)	Time spent in closed arms (s)
Control	10 mL/kg	131.27 ± 1.71	168.73 ± 1.71
Diazepam	1	224.70 ± 2.34	75.21 ± 2.37
CEF‐ME	200	142.33 ± 1.73	157.67 ± 1.73
CEF‐ME	400	172.47 ± 2.25	127.53 ± 2.25***

*Note:* Statistical significance levels were denoted as follows: *p < 0.05, **p < 0.01, and ***p < 0.001, in comparison to the control group.

#### 3.2.2 Hole‐Board Test

**FIGURE 4:**
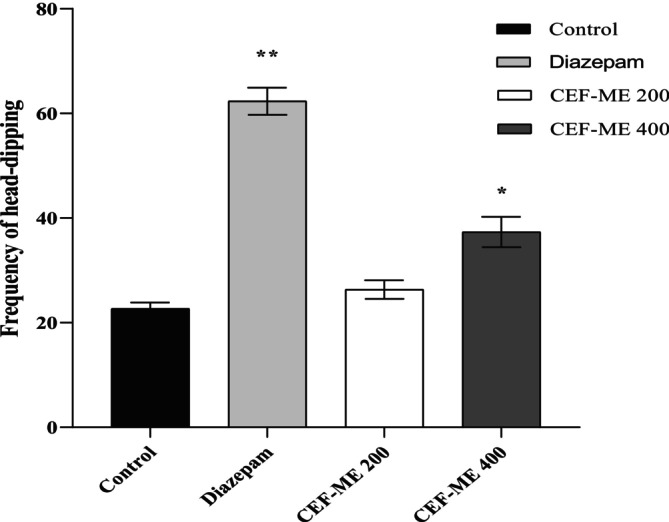
Evaluation of anxiolytic activity of CEF‐ME by using HBT. CEF‐ME = *Colocasia esculenta* flowers methanol extract.

Table 7 and Figure 4 show the conventional diazepam and sample solution 200 and 400 mg/kg dose head‐dipping frequency.

**TABLE 7:** Effects of CEF‐ME on mice in the Hole‐Board apparatus. CEF‐ME = *Colocasia esculenta* flowers methanol extract.

Sample	Dose (mg/kg)	Frequency of head‐dipping
		Mean ± SEM
Control	10 ml/kg	22.67 ± 1.2
Diazepam	1	62.33 ± 2.6
CEF‐ME	200	26.33 ± 1.76
CEF‐ME	400	37.33 ± 2.91

*Note:* Statistical significance levels were denoted as follows: *p < 0.05, **p < 0.01, and ***p < 0.001, in comparison to the control group.

#### 3.2.3 Light–Dark Test

**FIGURE 5:**
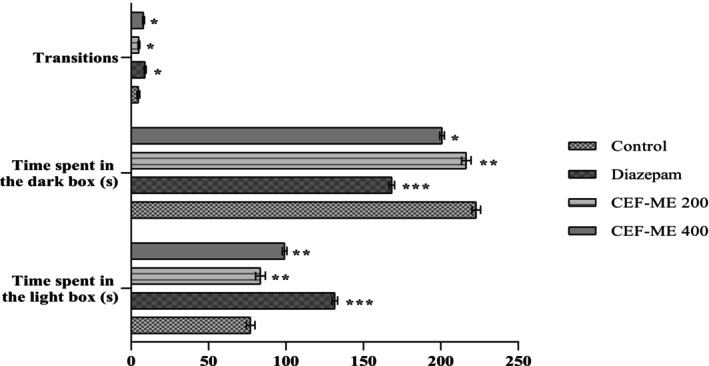
Evaluation of anxiolytic activity of CEF‐ME using LDT. CEF‐ME = *Colocasia esculenta* flowers methanol extract.

CEF‐ME exhibited significant anxiolytic activity in the light–dark test (Table 8 and Figure 5).

**TABLE 8:** Effects of CEF‐ME on mice in the light–dark test. CEF‐ME = *Colocasia esculenta* flowers methanol extract.

Sample	Dose (mg/kg)	Time spent in the light box (s)	Time spent in the dark box (s)	Transitions
Control	10	77.13 ± 2.82	222.87 ± 2.83	4.67 ± 0.88
Diazepam	1	131.60 ± 1.78	168.40 ± 1.78	9 ± 0.58
CEF‐ME	200	83.53 ± 3.12	216.47 ± 3.12	5 ± 0.58
CEF‐ME	400	99.20 ± 1.50	200.8 ± 1.50	8 ± 0.58

*Note:* The values displayed are given as mean ± SEM. Statistical significance levels were denoted as follows: *p < 0.05, **p < 0.01, and ***p < 0.001, in comparison to the control group.

### 3.3 Sedative Activity

#### 3.3.1 Open‐Field Test

**FIGURE 6:**
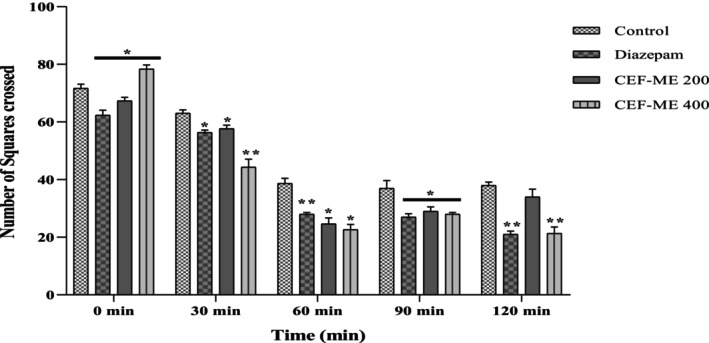
Evaluation of sedative activity of CEF‐ME by using the open‐field test. CEF‐ME = *Colocasia esculenta* flowers methanol extract. Statistical significance levels were denoted as follows: *p < 0.05, **p < 0.01, and ***p < 0.001, in comparison to the control group.

According to open‐field data, CEF‐ME demonstrated a substantial sedative effect (Table 9, Figure 6).

**TABLE 9:** Effects of CEF‐ME on mice in the open‐field test. CEF‐ME = *Colocasia esculenta* flowers methanol extract.

Treatment dose (mg/kg)	Number of squares crossed				
	0 min	30 min	60 min	90 min	120 min
Control	71.67 ± 1.45	63 ± 1.15	38.67 ± 1.76	37 ± 2.65	38 ± 1.15
Diazepam	62.33 ± 1.76	56.33 ± 0.88	28 ± 0.58	27 ± 1.15	21 ± 1.15
CEF‐ME 200	67.33 ± 1.2	57.67 ± 1.2	24.67 ± 2.03	29 ± 1.53	34 ± 2.65
CEF‐ME 400	78.33 ± 1.45	44.33 ± 2.73	22.67 ± 1.76	28 ± 0.58	21.33 ± 2.19

*Note:* The values displayed are given as Mean ± SEM. Statistical significance levels were denoted as follows: *p < 0.05, **p < 0.01, and ***p < 0.001, in comparison to the control group.

#### 3.3.2 Hole‐Cross Test

**FIGURE 7:**
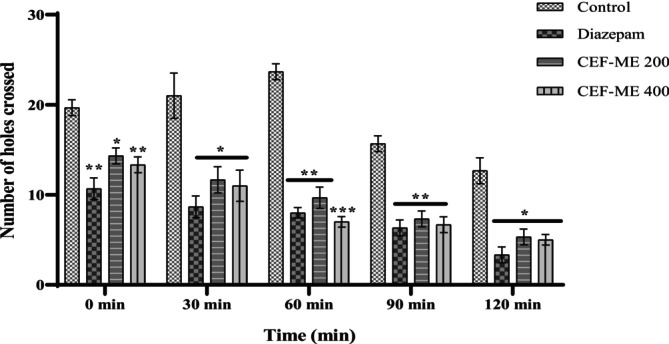
Sedative Activity of CEF‐ME by using the Hole‐Cross Test. CEF‐ME = *Colocasia esculenta* flowers methanol extract. Statistical significance levels were denoted as follows: *p < 0.05, **p < 0.01, and ***p < 0.001, in comparison to the control group.

As seen in Table 10 and Figure 7, there was a progressive decrease in the number of mice that crossed between the chambers during the 2‐h Hole‐Cross Test.

**TABLE 10:** Effects of CEF‐ME on mice in hole‐Cross Test. CEF‐ME = *Colocasia esculenta* flowers methanol extract.

Treatment dose (mg/kg)	Number of holes crossed				
	0 min	30 min	60 min	90 min	120 min
Control	19.67 ± 0.88	21 ± 2.52	23.67 ± 0.88	15.67 ± 0.88	12.67 ± 1.45
Diazepam	10.67 ± 1.20	8.67 ± 1.20	8 ± 0.58	6.33 ± 0.88	3.33 ± 0.88
CEF‐ME 200	14.33 ± 0.88	11.67 ± 1.45	9.67 ± 1.20	7.33 ± 0.88	5.33 ± 0.88
CEF‐ME 400	13.33 ± 0.88	11 ± 1.73	7 ± 0.58	6.67 ± 0.88	5 ± 0.58

*Note:* The values displayed are given as mean ± SEM. Statistical significance levels were denoted as follows: *p < 0.05, **p < 0.01, and ***p < 0.001, in comparison to the control group.

### 3.4 Antidepressant Activity

#### 3.4.1 Forced‐Swimming Test

**FIGURE 8:**
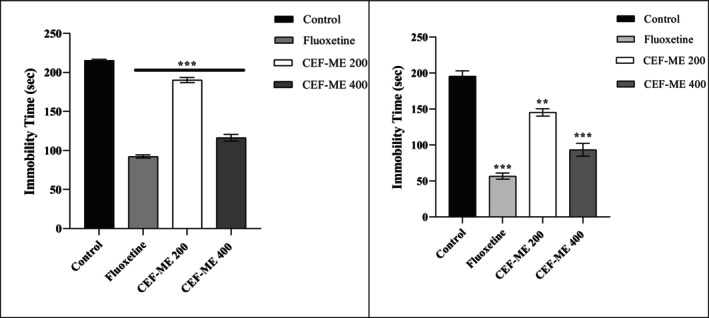
Antidepressant Activity of CEF‐ME by using FST (left) and TST (right). CEF‐ME = *Colocasia esculenta* flowers methanol extract. Statistical significance levels were denoted as follows: *p < 0.05, **p < 0.01, and ***p < 0.001, in comparison to the control group.

The FST results are summarized in Table 11 and Figure 8.

**TABLE 11:** Effects of CEF‐ME on mice in Forced‐Swimming Test. CEF‐ME = *Colocasia esculenta* flowers methanol extract.

Sample (mg/kg)	Immobility time					
	G‐1	G‐2	G‐3	Mean	SEM	*p*
Control	213	218	215	215.33	1.45	—
Fluoxetine (25)	96	89	92	92.33	2.03	0.000
CEF‐ME 200	185	190	196	190.33	3.18	0.000
CEF‐ME 400	110	125	114	116.33	4.48	0.000

*Note:* The values displayed are given as mean ± SEM. Statistical significance levels were denoted as follows: *p < 0.05, **p < 0.01, and ***p < 0.001, in comparison to the control group.

#### 3.4.2 Tail‐Suspension Test

The TST results are presented in Table 12 and Figure 8.

**TABLE 12:** Effects of CEF‐ME on mice in the Tail‐Suspension Test. CEF‐ME = *Colocasia esculenta* flowers methanol extract.

Sample (mg/kg)	Immobility time		
	Mean	SEM	*p*
Control	195.67	7.45	—
Fluoxetine (25)	56.67	4.26	0.000
CEF‐ME 200	145.33	5.21	0.001
CEF‐ME 400	93.33	8.82	0.000

*Note:* The values displayed are given as mean ± SEM. Statistical significance levels were denoted as follows: *p < 0.05, **p < 0.01, and ***p < 0.001, in comparison to the control group.

### 3.5 In Silico Study

**FIGURE 9:**
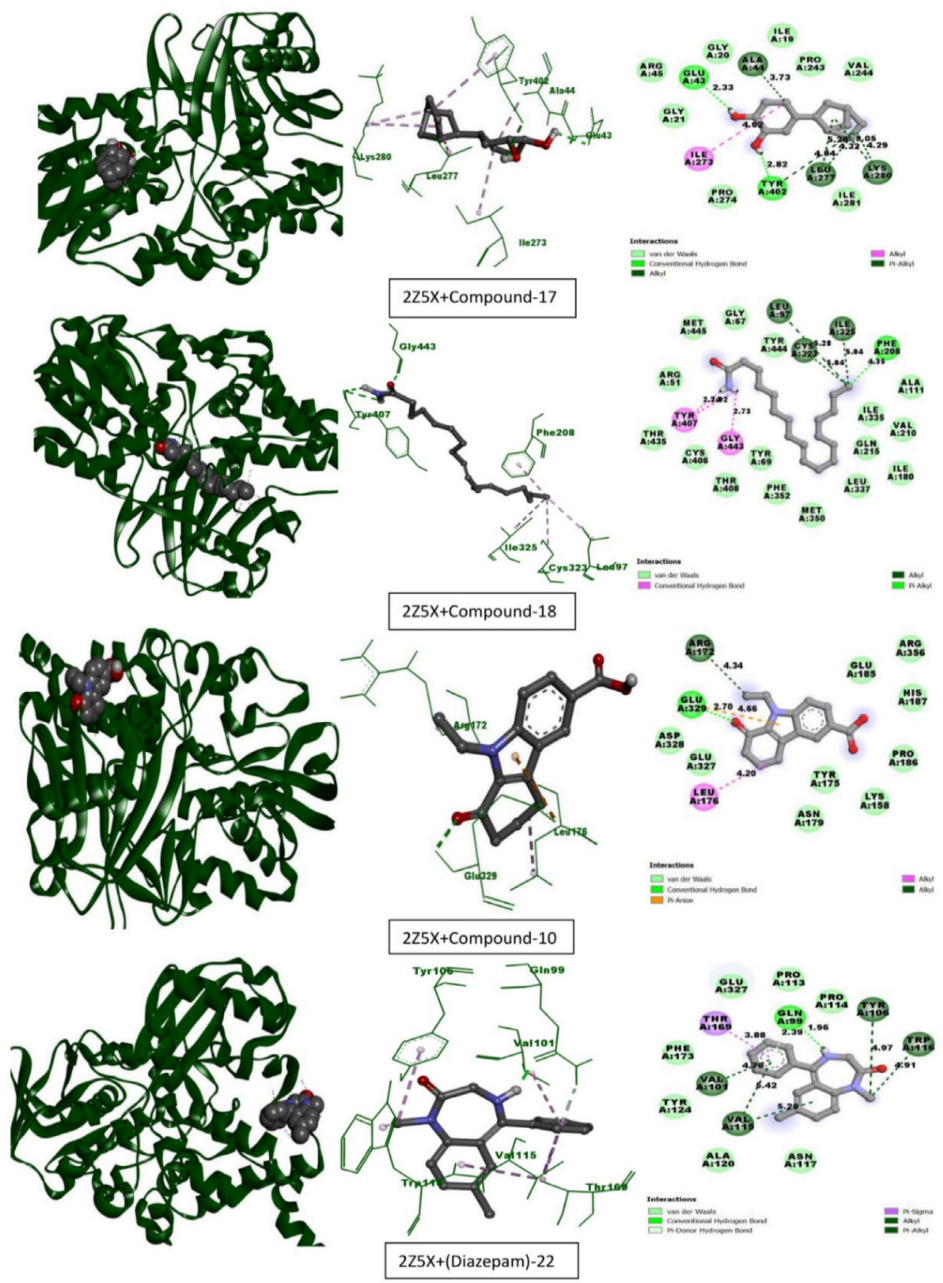
Molecular docking interactions of top‐docked compounds and standard with the human monoamine oxidase A (PDB ID: 2Z5X).

**FIGURE 10:**
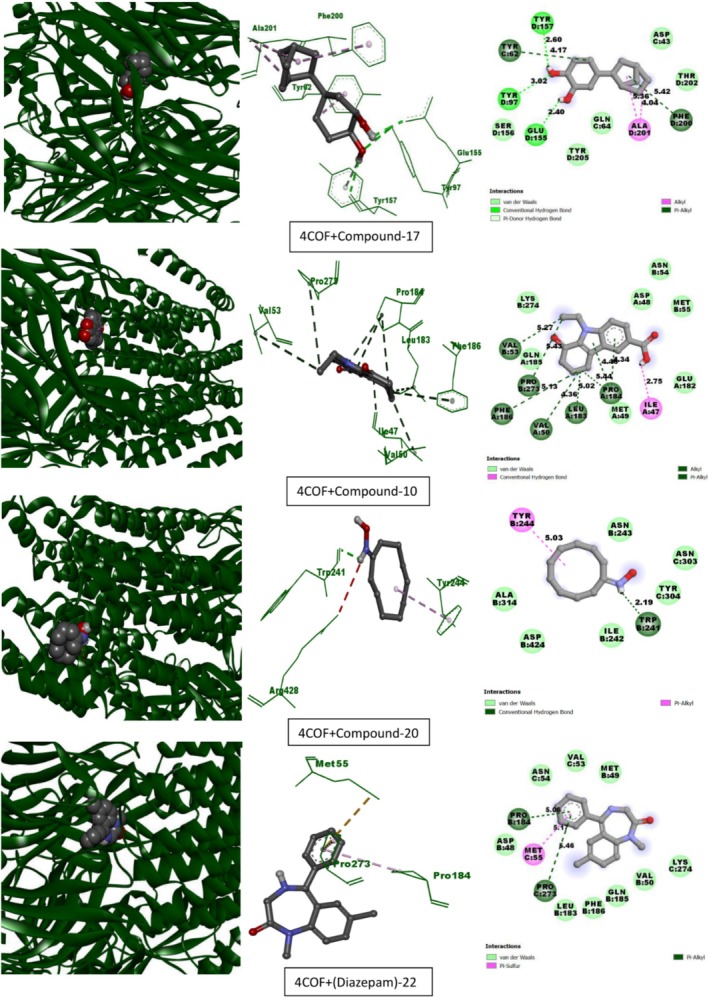
Molecular docking interactions of top‐docked compounds and standard with the gamma‐aminobutyric acid receptor (PDB ID: 4COF).

**FIGURE 11:**
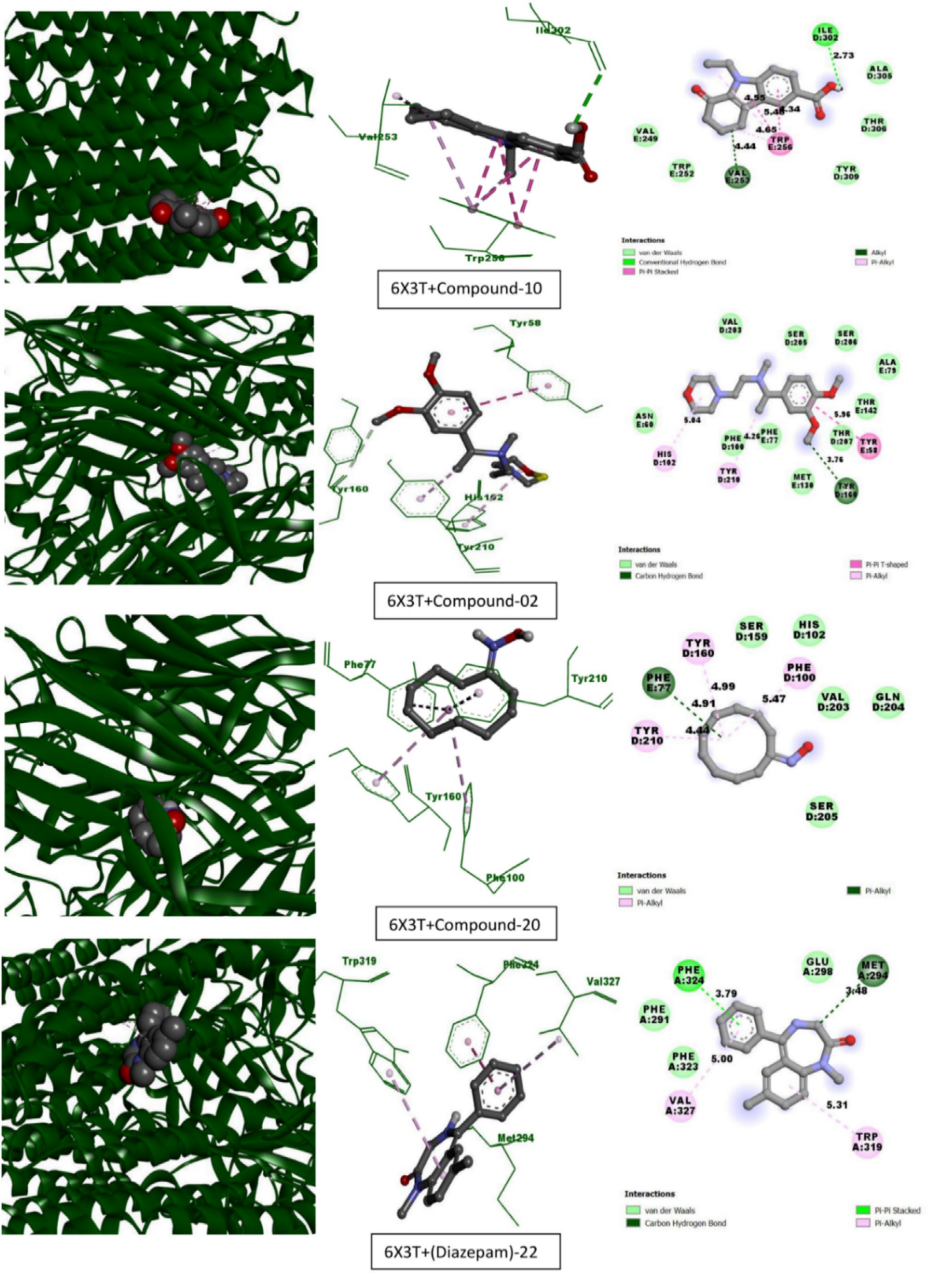
Molecular docking interactions of top‐docked compounds and standard with the human GABA_A_ receptor alpha1‐beta2‐gamma2 subtype (PDB: 6X3T).

**FIGURE 12:**
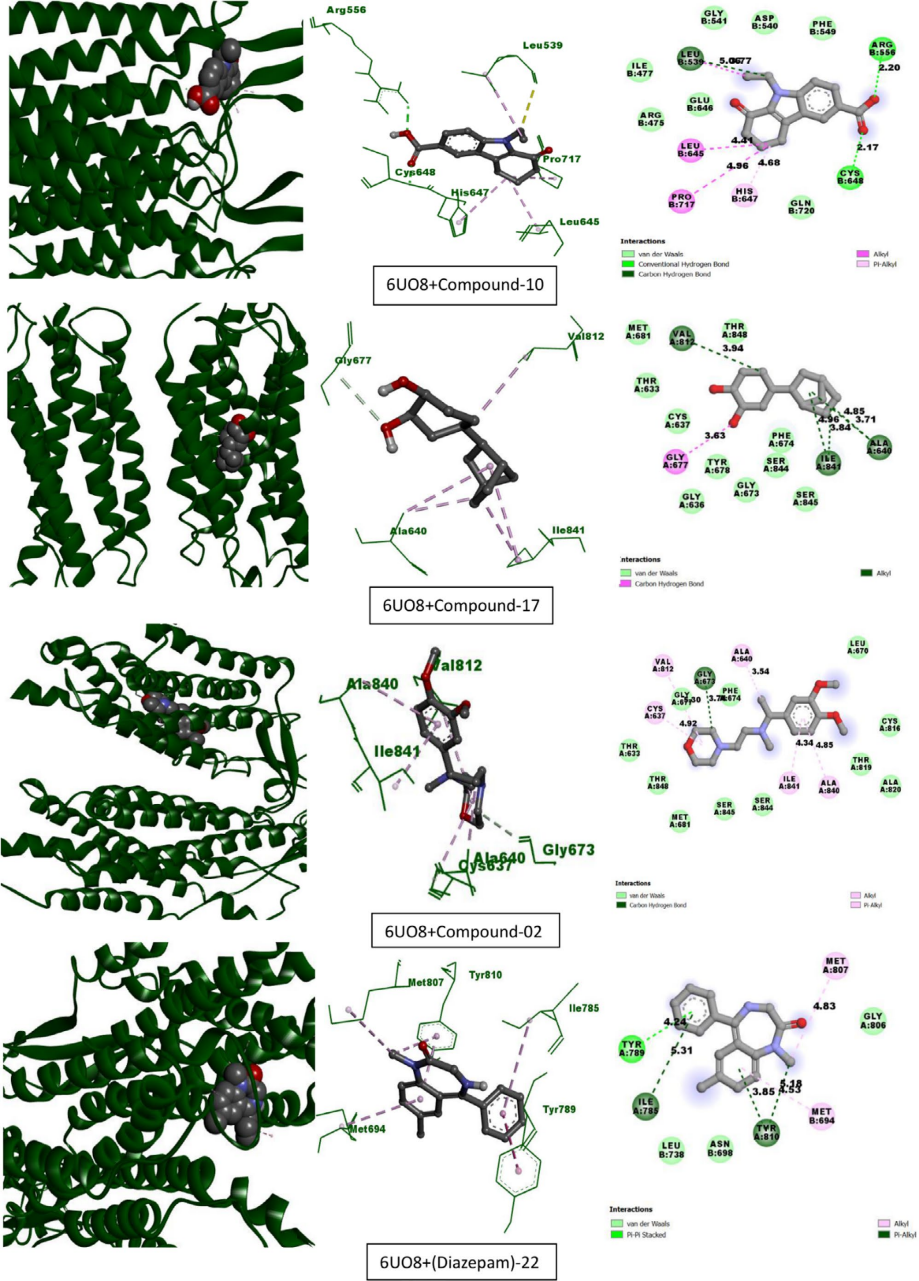
Molecular docking interactions of top‐docked compounds and standard with the human metabotropic GABA(B) receptor (PDB: 6UO8).

**FIGURE 13:**
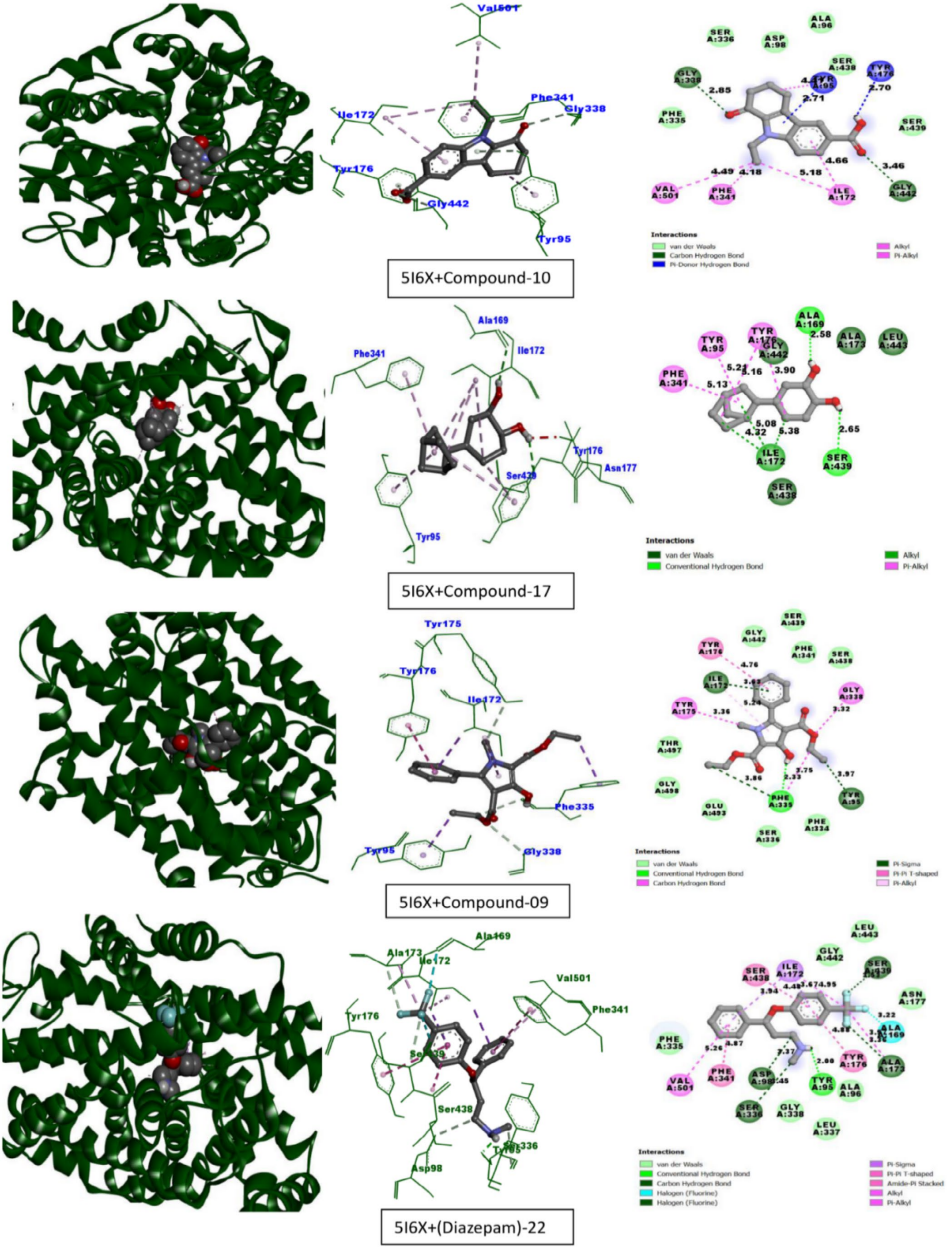
Molecular docking interactions of top‐docked compounds and standard with the paroxetine‐human serotonin transporter complex (PDB: 5I6X).

**FIGURE 14:**
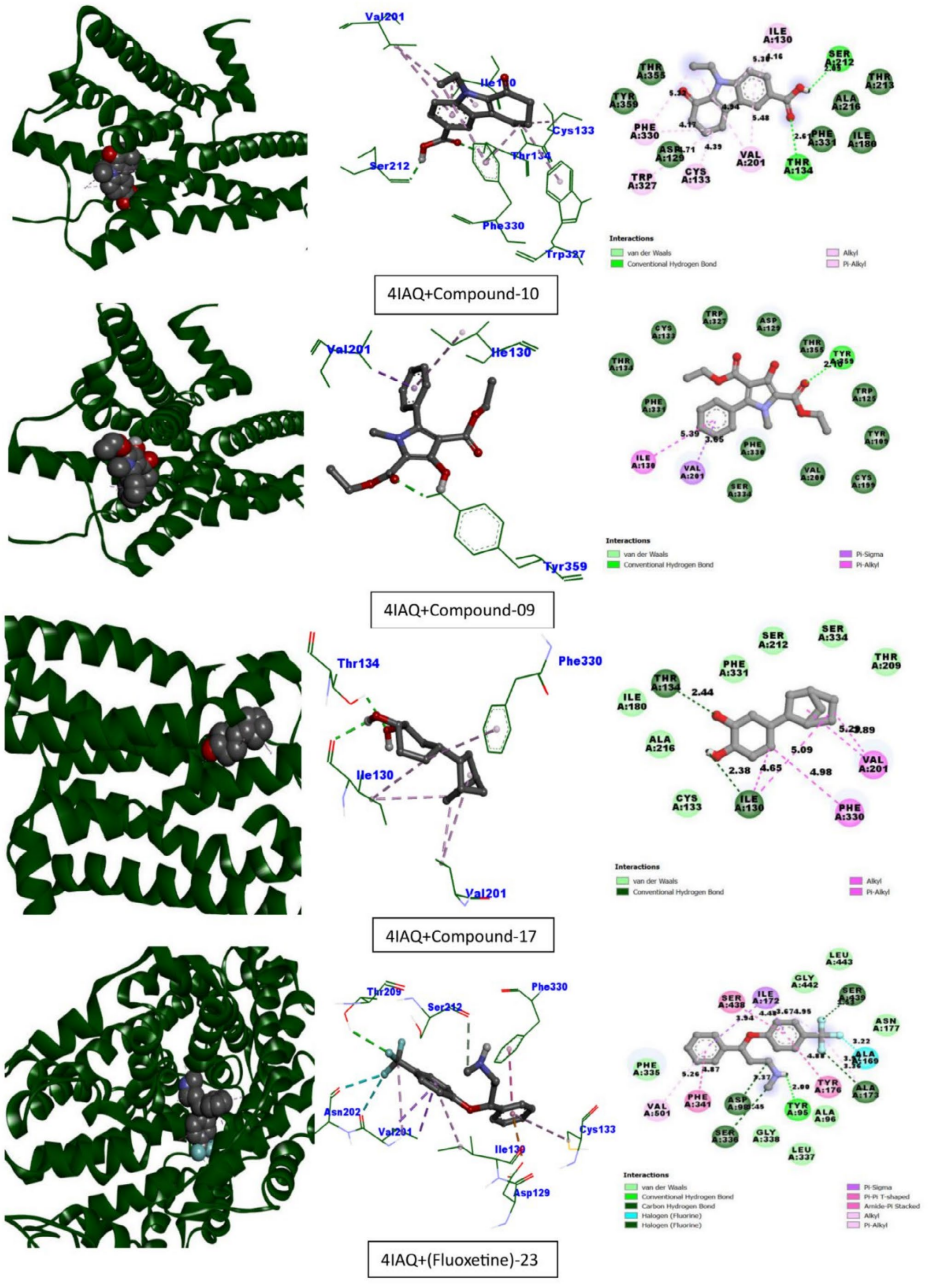
Molecular docking interactions of top‐docked compounds and standard with the chimeric protein of 5‐HT1B‐BRIL (PDB: 4IAQ).

Table 13 lists the compounds from CEF‐ME and their docking scores against various receptors, including GABA_A_, monoamine oxidase A, serotonin transporter, 5‐HT1B‐BRIL, and GABA (B) receptors. The interaction of the compounds from CEF‐ME with the amino acid residues of selected proteins is illustrated in Table 14 and Figures 9, 10, 11, 12, 13, 14. These figures provide detailed insights into the molecular docking analysis, showcasing how the bioactive compounds in CEF‐ME bind to the active sites or other critical regions of the target proteins.

**TABLE 13:** The docking score of the selected compounds identified from the CEF‐ME. CEF‐ME = *Colocasia esculenta* flowers methanol extract.

Sl no.	Compounds	Sedative		Anxiolytic		Antidepressant	
		6X3T	6UO8	2Z5X	4COF	5I6X	4IAQ
1	3‐Allyloxy‐1,2 propanediol_78950	−4.5	−4.1	−4.6	−4.1	−4.7	−4.4
2	Ethanamine, 1‐(3,4‐dimethoxyphenyl)‐N‐methyl‐N‐[2‐(4‐morpholyl)ethyl]_585380	−7.3	−6.6	−6.5	−6.4	−6.7	−6.8
3	6‐Oxa‐bicyclo[3.1.0]hexan‐3‐one _535532	−4.7	−4.3	−4.9	−4.5	−5	−4.5
4	Propane, 1,1‐diethoxy‐2‐methyl‐519415	−4.8	−4.4	−4.3	−4.9	−4.8	−4.2
5	2‐Propen‐1‐ol‐7858	−4.2	−3.1	−3.5	−3.4	−3.6	−3.1
6	Piperidine, 1‐nitroso‐7526	−5.1	−4.7	−5.7	−5.7	−4.9	−5
7	d‐Gala‐l‐ido‐octonic amide_552061	−6.5	−5.1	−7.1	−6.1	−5.6	−5.9
8	5‐Methoxy‐3‐phenyl‐1‐pentanol‐542519	−6.4	−5.6	−6.2	−6.5	−6.4	−6.1
9	Diethyl 1‐methyl‐3‐hydroxy‐5‐phenylpyrrole‐2,4‐dicarboxylate_582209	−6.4	−6.5	−7.1	−6.1	−7.7	−7.5
10	3,3′‐Thiodipropanol_768661	−7.6	−7.2	−7.2	−7.4	−8	−8.5
11	Phenol, 2,6‐dimethoxy_7041	−6.3	−5	−5.6	−5.5	−5.4	−5.1
12	2‐Furanmethanol, 5‐ethenyltetrahydro‐alpha,alpha,5‐trimethyl‐22310	−6.4	−6.1	−6.2	−5.4	−6.4	−5.9
13	2‐(Isobutoxymethyl)oxirane‐98155	−4.8	−4.3	−5.1	−4.6	−4.3	−4.4
14	3,3‐Dimethylpentanoic acid‐5282645	−4.9	−4.8	−5.6	−5.3	−5	−4.9
15	Octyl‐.beta.‐D‐glucopyranoside‐62852	−5.8	−6.1	−6.2	−6.1	−6.9	−6.6
16	3‐Deoxy‐d‐mannoic lactone_10350	−5.7	−5.2	−6.1	−5.3	−5.2	−5.3
17	Cyclohexane‐1,2‐diol, 4‐(bicylo[2.2.1]hept‐2‐yl)_535167	−6.4	−7.1	−8.5	−8.1	−8	−7.3
18	9‐Octadecenamide_5283387	−4.8	−5.8	−7.6	−5.9	−6.5	−5.9
19	Cyclohexanecarboxamide‐14283	−6.4	−5.5	−6.1	−6.5	−5.9	−5.6
20	Cyclodecanone, oxime_76303	−7.2	−6.4	−6.5	−6.6	−7.5	−6.8
*Standard*							
21	Diazepam_3016	−7.7	−7.9	−8.2	−7.5	—	—
22	Fluoxetine_3386					−9.1	−8.1

**TABLE 14:** CEF‐ME's selected phytochemicals *in silico* binding affinity and non‐bonding interaction for anxiolytic (2Z5X, 4COF), sedative (6X3T, 6UO8), and antidepressant (5I6X, 4IAQ) properties, respectively.

Section no	Receptor	Compound	Binding affinity (kcal/mol)	Bond type	Amino acids
1	2Z5X	17	−8.5	Conventional hydrogen bond	TYR402, GLU43
				Alkyl	ALA44, LEU277, LYS280, ILE273, LEU277, LYS280
				Pi‐Alkyl	TYR402
		18	−7.6	Conventional hydrogen bond	TYR407, TYR407, GLY443
				Alkyl	LEU97, CYS323, ILE325
				Pi‐Alkyl	PHE208
		10	−7.2	Conventional hydrogen bond	GLU329
				Pi‐Anion	GLU329
				Alkyl	LEU176, ARG172
		Diazepam	−8.2	Conventional hydrogen bond	GLN99
				Pi‐donor hydrogen bond	GLN99
				Pi‐Sigma	THR169
				Alkyl	VAL115
				Pi‐Alkyl	TYR106, TRP116, VAL101, VAL115
2	4COF	17	−8.1	Conventional hydrogen bond	TYR97, GLU155, TYR157
				Pi‐donor hydrogen bond	TYR157
		10	−7.4	Alkyl	ALA201, ALA201
				Pi‐Alkyl	TYR62, PHE200
		20	−6.6	Conventional hydrogen bond	ILE47
				Alkyl	VAL50, LEU183, PRO184, VAL53, PRO273
				Pi‐Alkyl	APHE186, PRO184, PRO184
		17	−8.1	Conventional hydrogen bond	TRP241
				Pi‐Alkyl	TYR244
		Diazepam	−7.5	Pi‐Sulfur	MET55
				Pi‐Alkyl	PRO184, PRO273
3	6X3T	10	−7.6	Conventional hydrogen bond	ILE302
				Pi‐Pi stacked	TRP256, TRP256, TRP256, TRP256
				Alkyl	VAL253
				Pi‐Alkyl	TRP256, TRP256
		2	−7.3	Carbon–hydrogen bond	TYR160
				Pi‐Pi T‐shaped	TYR58
				Pi‐Alkyl	HIS102, TYR210
		20	−7.2	Pi‐Alkyl	PHE100, TYR160, TYR210, PHE77
		Diazepam	−7.7	Carbon–hydrogen bond	MET294
				Pi‐Pi stacked	PHE324
				Pi‐Alkyl	TRP319, VAL327
4	6UO8	10	−7.2	Conventional hydrogen bond	ARG556, CYS648
				Carbon–hydrogen bond	LEU539
				Alkyl	LEU645, PRO717, LEU539
				Pi‐Alkyl	HIS647
		17	−7.1	Carbon–hydrogen bond	GLY677
				Alkyl	ALA640, ALA640, ILE841, VAL812, ILE841
		9	−6.6	Carbon hydrogen	GLY673:O
				Alkyl	CYS637, ALA640, VAL812
				Pi‐Alkyl	ALA840, ILE841
		Diazepam	−7.9	Pi‐Pi stacked	TYR789
				Alkyl	MET694, MET807
				Pi‐Alkyl	TYR810, TYR810, ILE785
5	5I6X	10	−8	Carbon–hydrogen bond	GLY338, GLY442
				Pi‐donor hydrogen bond	TYR95, TYR176
				Alkyl	ILE172, VAL501
				Pi‐Alkyl	TYR95, PHE341, ILE172
		17	−8	Conventional hydrogen bond	ALA169, SER439
				Alkyl	ILE172, ILE172, ILE172
				Pi‐Alkyl	TYR95, TYR176, TYR176, PHE341
		9	−7.7	Conventional hydrogen bond	PHE335
				Carbon–hydrogen bond	GLY338, TYR175, PHE335
				Pi‐Sigma	ILE172, TYR95, PHE335
				Pi‐Pi T‐shaped	TYR176
				Pi‐Alkyl	ILE172
		Fluoxetine	−9.1	Conventional hydrogen bond	TYR95
				Carbon–hydrogen bond	ALA173, SER439, ASP98, SER336
				Halogen (Fluorine)	ALA169, SER439
				Pi‐Sigma	ILE172, ILE172
				Pi‐Pi T‐shaped	TYR176, PHE341
				Amide‐Pi Stacked	SER438, SER439
				Alkyl	ALA173
				Pi‐Alkyl	ILE172
				Carbon–hydrogen bond	VAL501
6	4IAQ	10	−8.5	Conventional hydrogen bond	THR134, SER212
				Alkyl	CYS133, VAL201
				Pi‐Alkyl	TRP327, PHE330, PHE330, ILE130, ILE130, VAL201
		9	−7.5	Conventional hydrogen bond	TYR359
				Pi‐Sigma	VAL201
				Pi‐Alkyl	ILE130
		17	−7.3	Conventional hydrogen bond	THR134, ILE130
				Alkyl	VAL201, ILE130, ILE130, VAL201
				Pi‐Alkyl	PHE330
		Fluoxetine	−8.1	Conventional hydrogen bond	THR209
				Carbon–hydrogen bond	SER212
				Halogen (Fluorine)	VAL201, ASN202
				Pi‐Anion	ASP129, VAL201, VAL201
				Pi‐Pi stacked	PHE330
				Alkyl	VAL201
				Pi‐Alkyl	ILE130, CYS133

#### 3.5.1 In Silico Molecular Docking Simulation for Anxiolytic Activities

This molecular docking study used Diazepam as the reference standard to assess how specific compounds interacted with two important receptors: the gamma‐aminobutyric acid receptor (PDB ID: 4COF) and the human monoamine oxidase A (PDB ID: 2Z5X). The objective was to identify possible anxiolytic substances with similar or better interaction patterns and binding affinities (Table 14, Sections 1 and 2, and Figures 9 and 10).

The maximum binding affinity was shown by Compound 17 (−8.5 kcal/mol) in contrast to Diazepam (−8.2 kcal/mol). The interactions involved extensive hydrophobic contacts with residues like ALA44 and LEU277, as well as hydrogen bonds with TYR402 and GLU43. These connections imply excellent specificity and stability of the receptor. Diazepam demonstrated potent interactions that confirmed its effectiveness, such as hydrogen bonding with GLN99 and pi‐alkyl interactions with TYR106 and TRP116. Compound 17 may be a more effective inhibitor of monoamine oxidase A, as evidenced by its improved binding profile.

The binding affinities of other molecules, including 18 and 10, were moderate (−7.6 and −7.2 kcal/mol, respectively). Despite having weaker interactions, they had noteworthy characteristics that suggested room for improvement, such as hydrogen bonds (compound 18 with TYR407 and GLY443) and Pi–Anion bonding (compound 10 with GLU329).

Compound 17 demonstrated a binding affinity of −8.1 kcal/mol for the gamma‐aminobutyric acid receptor, which was equivalent to the interaction strength of diazepam. It showed a robust and varied network of contacts by forming hydrogen bonds with TYR97 and GLU155, as well as alkyl interactions with ALA201 and Pi‐Donor hydrogen bonds with TYR157. With notable interactions including Pi‐Sulfur bonding with MET55 and Pi‐Alkyl interactions with residues like PRO184 and PRO273, diazepam demonstrated a binding affinity of −7.5 kcal/mol, confirming its well‐established anxiolytic function.

The binding affinities of other compounds, such as 10 and 20, were lower (−7.4 and −6.6 kcal/mol, respectively). Nonetheless, these substances exhibited significant interactions, including hydrophobic and conventional hydrogen bonds, suggesting moderate receptor binding and the need for further research.

#### 3.5.2 In Silico Molecular Docking Simulation for Sedative Activities

This molecular docking study used Diazepam as the reference standard to assess how specific compounds interacted with two important receptors: the human GABA_A_ receptor alpha1‐beta2‐gamma2 subtype (PDB: 6X3T) and the human metabotropic GABA(B) receptor (PDB: 6UO8) to their sedative effects (Table 14, Sections 3 and 4, and Figures 11 and 12).

Compound 10 has a binding affinity of −7.6 kcal/mol for the GABA_A_ receptor (6X3T), which is highly similar to that of diazepam, with a binding affinity of −7.7 kcal/mol. Compound 10 exhibits strong Pi‐Pi stacking interactions with TRP256 and a hydrogen bond with ILE302. The binding affinities for compounds 2 and 20 were −7.3 kcal/mol and −7.2 kcal/mol, respectively.

Compound 10's binding affinity for the GABA(B) receptor (6UO8) is −7.2 kcal/mol, which is competitive even though it is less than that of diazepam (−7.9 kcal/mol). With ARG556 and CYS648, compound 10 forms hydrogen bonds; with HIS647, it forms pi‐alkyl interactions. A binding affinity of −7.1 kcal/mol was demonstrated by compound 17 and −6.6 kcal/mol by compound 9.

Compound 10 demonstrated robust interactions with the GABA(B) receptor (−7.2 kcal/mol). It closely matched the performance of Diazepam with the GABAA receptor (−7.6 kcal/mol), making it a promising option for further research. Compound 17 also demonstrated a steady affinity for binding, especially with the GABA(B) receptor, indicating room for improvement. Compound 9 required an improvement in its structure to enhance receptor interaction, as it exhibited only modest activity.

#### 3.5.3 *In Silico* Molecular Docking Simulation for Antidepressant Activities

This molecular docking study used Fluoxetine as the reference standard to assess how specific compounds interacted with two important receptors: the 5‐HT1B‐BRIL receptor (PDB: 4IAQ) and the paroxetine‐human serotonin transporter complex (PDB: 5I6X), regarding their antidepressant effects (Table 14, Sections 5 and 6, and Figures 13 and 14).

Compounds 10 and 17 exhibited similar binding affinities of −8 kcal/mol with the 5I6X receptor, indicating strong receptor engagement. Compound 10 formed hydrophobic Pi‐Alkyl interactions (PHE341) and Pi‐Donor hydrogen bonds (TYR95 and TYR176). Similar to fluoxetine, compound 17 also exhibited substantial Pi‐Alkyl interactions and hydrogen bonding (ALA169, SER439). Conversely, fluoxetine exhibited a binding affinity of −9.1 kcal/mol. The binding affinity of compound 9 was −7.7 kcal/mol, which was somewhat lower than that of the other compounds.

With a binding affinity of −8.5 kcal/mol, compound 10 demonstrated superiority over fluoxetine with the 4IAQ receptor. It generated hydrophobic Pi‐Alkyl contacts with PHE330 and TRP327, as well as strong hydrogen bonds (THR134, SER212). A binding affinity of −8.1 kcal/mol was shown for fluoxetine. Their binding affinities were −7.5 kcal/mol for compound 9 and −7.3 kcal/mol for compound 17.

Compound 10 was a promising candidate for further research, as it demonstrated a similar or even higher binding affinity, particularly with the 4IAQ receptor. Strong hydrogen bonds and a wide range of hydrophobic interactions suggest that it may be used to target receptors effectively. Since compound 10 exhibits better or equivalent binding affinities and interacts with key residues in both receptors, it shows great promise as a substitute for fluoxetine, a highly effective antidepressant. Compound 17 also shows promise, performing consistently, whereas compound 9 may require modification to enhance its effectiveness. To confirm the therapeutic potential of compounds 10 and 17, these results warrant further in vitro and in vivo research.

## 4 Discussion

The herb *C. esculenta* Linn. (family: Araceae), commonly known as taro, has been widely used in traditional medicine across tropical and subtropical regions for treating various ailments like skin conditions, neurological disorders, internal hemorrhages, asthma, arthritis, and diarrhea (Prajapati et al. 2011). In this study, an acute toxicity study was conducted to evaluate the general behavior and physical condition of the mice. No mortality was recorded at the 2000 mg/kg dose. We compared the general appearance and behavioral characteristics of control and treated groups, noting that the control group maintained standard physiological and behavioral patterns, such as typical growth and grooming behaviors. Deviations from this in the treated group could indicate potential adverse effects. If no significant differences were observed, it could imply that the treatment had no harmful or beneficial effects. However, we recognize that the lack of data from long‐term or chronic exposure restricts a comprehensive assessment of the safety margin.

The study further explored the organ‐to‐body weight index in mice after administering the methanolic extract of *C. esculenta* flowers (CEF‐ME). Results showed a significant increase in the organ weight ratios, especially in the liver, suggesting a possible inflammatory or adaptive response (Dahamna et al. 2011a; Hayelom et al. 2012). A notable rise in liver weight, from 5.44% in the control group to 8.81% in the treated group, indicated that the liver might be susceptible to CEF‐ME. The increases in organ weight, such as in the kidney, lung, spleen, and heart, point to a systemic effect, potentially due to heightened metabolic activity or stress caused by the extract (Abou et al. 2015; Martey et al. 2013). Further histopathological and biochemical studies are necessary to understand these changes and determine whether they reflect toxicity or adaptive responses (Dahamna et al. 2011; Ono et al. 1988).

Biochemical analysis revealed significant alterations in liver and kidney function, as well as lipid metabolism. Transaminases such as AST and ALT are critical markers of liver function and potential damage. In this study, a decrease in ALT levels in the treatment group suggests a hepatoprotective effect of CEF‐ME (Al‐kareem et al. 2020). However, elevated AST levels may point to hepatic or muscular damage (Ikitde et al. 2015). Similarly, the reduction in ALP levels may reflect improved liver function (N. Jain et al. 2012). Increased creatinine levels in the treated group indicated potential nephrotoxic effects at higher doses, requiring further investigation (N. Jain et al. 2012).

Additionally, lipid metabolism was significantly affected by CEF‐ME, with the treatment group showing lower cholesterol and triglyceride levels, indicating potential hypolipidemic effects (Nguyen et al. 2019). The drop in HDL levels, however, could counteract some cardiovascular benefits (Nguyen et al. 2019). These findings underscore the complexity of the extract's effects, which may encompass both therapeutic benefits and potential risks. Further studies are essential to examine the long‐term effects of CEF‐ME and its dose‐dependent impact on organ function (Nguyen et al. 2019).

Hematological analysis also revealed notable changes. The treatment group showed increased white blood cell (WBC) count, suggesting an immune response (Dicato 2008), while differential counts indicated an inflammatory reaction, with higher neutrophil and monocyte levels and lower lymphocyte percentages (Gerds et al. 2022). The increased RBC count and hemoglobin levels in the treated group suggested that CEF‐ME may stimulate erythropoiesis (Gallagher et al. 2022), while the decreased platelet count pointed to potential thrombocytopenia (Ahmadinejad et al. 2017). These changes highlight both immune‐modulatory effects and possible adverse effects, underscoring the need for careful dose management and further research (Gerds et al. 2022).

In vivo models like the Elevated Plus Maze (EPM), Hole‐Board Test (HBT), and Light‐Dark Box Test (LDT) demonstrated that CEF‐ME might have minor anxiolytic effects, as evidenced by changes in animals' open‐arm behavior and head‐dipping activity (Nurfitria et al. 2015; Zhang and Yao 2019). These preliminary findings suggest that the extract may interact with the GABAergic system, potentially through GABA receptors, to exert anxiolytic effects (Nkwemeh et al. 2023). However, these effects were more pronounced at higher doses, and further studies are needed to elucidate the precise mechanisms involved.

Depression‐related behaviors were assessed using the Forced‐Swimming Test (FST) and Tail‐Suspension Test (TST) (Borah et al. 2016) Both tests showed that CEF‐ME significantly reduced immobility times in a dose‐dependent manner, indicating antidepressant‐like effects (Ahmed et al. 2009; Hemby et al. 1997) The 400 mg/kg dose of CEF‐ME produced a more significant reduction in immobility, comparable to the effects of fluoxetine, suggesting that CEF‐ME may modulate neurochemical systems involved in mood regulation. This highlights the potential of CEF‐ME as an antidepressant alternative (Borah et al. 2016).

While the findings suggest that CEF‐ME has potential therapeutic effects, including anxiolytic and antidepressant‐like actions, caution is warranted due to possible hepatotoxic and nephrotoxic effects at higher doses. Further research is needed to investigate the mechanisms underlying these effects and to assess the safety and efficacy of CEF‐ME for clinical use. These results provide a foundation for future studies that could lead to the development of novel treatments for neurological disorders, including depression and anxiety, while emphasizing the importance of dose optimization and safety monitoring.

The therapeutic potential of *C. esculenta* appears promising; however, limitations such as small sample sizes and lack of study replication warrant more statistically rigorous investigations. Gene expression studies are crucial for identifying disease‐relevant molecular pathways and correlating pharmacodynamic effects with dosage. Some data suggest a dose‐dependent relationship; however, a comprehensive evaluation of the dose–response is necessary to accurately determine the most effective dosage range, with histopathological analysis providing critical insights. Furthermore, chemical profiling and extract standardization are crucial for ensuring consistency and efficacy. Importantly, as data from Swiss albino mice may not fully translate to human outcomes, further research using diverse animal models is imperative.

## 5 Conclusion

The present study evaluated the potential toxicological profile of the methanol extract of *C. esculenta* flowers through comprehensive biochemical, hematological, and behavioral assessments. *C. esculenta* is consumed as food in many countries, although its safety profile is dose‐dependent, as higher concentrations exhibit signs of toxicity. Biochemical analyses revealed the extract's potential to modulate enzymatic activities and oxidative stress markers, suggesting its utility in managing oxidative stress‐related conditions. Hematological assessments further supported the extract's biological activity, although adverse effects were observed at higher doses. Behavioral studies provided additional insights, highlighting potential toxic effects at toxic doses. Although the flowers of *C. esculenta* are commonly consumed as food in Bangladesh, the study revealed that they exhibit various poisonous effects when consumed in high doses. Overall, CEF‐ME flowers show promise as a source of bioactive compounds with therapeutic applications, but caution must be exercised to establish appropriate dosing regimens. Further studies, including chemical profiling and standardization of the methanolic extract, isolation of individual bioactive compounds, and clinical trials, are warranted to explore its pharmacological potential and ensure safety for therapeutic use.

## Author Contributions

**Mahathir Mohammad:** data curation (equal), investigation (equal), software (equal), visualization (equal), writing – original draft (equal). **Md. Tanvir Chowdhury:** investigation (equal), software (equal), writing – original draft (equal). **Nazmul Hasan Eshaque:** data curation (equal), investigation (equal), writing – original draft (equal). **Md. Jahirul Islam Mamun:** data curation (equal), investigation (equal), writing – original draft (equal). **Sayed Al Hossain Rabbi:** data curation (equal), investigation (equal), writing – original draft (equal). **Md. Amzad Hasan:** data curation (equal), investigation (equal), writing – original draft (equal). **Miton Chowdhury:** data curation (equal), investigation (equal). **Md. Al‐mamun:** data curation (equal), investigation (equal). **Md. Jabir Rashid:** data curation (equal), investigation (equal). **S. M. Moazzem Hossen:** conceptualization (lead), data curation (equal), formal analysis (lead), methodology (lead), software (equal), supervision (lead), writing – original draft (equal), writing – review and editing (lead).

## Disclosure

Declaration of Generative AI and AI‐Assisted Technologies in the Writing Process: AI and AI‐supported technologies were used exclusively for grammar correction and sentence structure simplification during the preparation of this article. The authors have meticulously reviewed and edited the content, and they take full accountability for its accuracy and overall quality.

## Conflicts of Interest

The authors declare no conflicts of interest.

## Data Availability

Data will be made available on request.
